# Applications of Adipose Tissue Micrografts (ATM) and Dermis Micrografts (DMG) in Wound Healing: A Scoping Review of Clinical Studies

**DOI:** 10.3390/bioengineering12090948

**Published:** 2025-08-31

**Authors:** Konstantinos Zapsalis, Orestis Ioannidis, Elissavet Anestiadou, Maria Pantelidou, Konstantinos Siozos, Christos Xylas, Georgios Gemousakakis, Angeliki Cheva, Chryssa Bekiari, Antonia Loukousia, Savvas Symeonidis, Stefanos Bitsianis, Manousos-Georgios Pramateftakis, Efstathios Kotidis, Ioannis Mantzoros, Stamatios Angelopoulos

**Affiliations:** 1 4th Department of Surgery, General Hospital “George Papanikolaou”, Aristotle University of Thessaloniki, 57010 Exochi, Greece; konstzapsalis@yahoo.gr (K.Z.); elissavetxatz@gmail.com (E.A.); kwstassiwzos@hotmail.gr (K.S.); x.xilas@gmail.com (C.X.); gtgeorge@rocketmail.com (G.G.); simeonidissavvas@yahoo.com (S.S.); sbitsiani@gmail.com (S.B.); mpramateftakis@hotmail.com (M.-G.P.); skotidis@gmail.com (E.K.); imanvol@gmail.com (I.M.); saggelopoulos@auth.gr (S.A.); 2 Department of Plastic and Reconstructive Surgery, James Paget University Hospitals NHS Foundation Trust, Gorleston, Great Yarmouth NR31 6LA, Norfolk, UK; mariapantelidou.el@hotmail.com; 3 Pathology Department, Faculty of Medicine, Aristotle University of Thessaloniki, 54124 Thessaloniki, Greece; antacheva@yahoo.gr (A.C.); tonialoukousia@gmail.com (A.L.); 4 Experimental and Research Center, Papageorgiou General Hospital of Thessaloniki, 56403 Thessaloniki, Greece; chmpekia@vet.auth.gr; 5 Laboratory of Anatomy and Histology, Veterinary School, Aristotle University of Thessaloniki, 54124 Thessaloniki, Greece

**Keywords:** adipose tissue micrografts, dermis micrografts, regenerative medicine, wound healing, autologous grafts, stromal vascular fraction, Rigenera system, tissue regeneration

## Abstract

Adipose tissue micrografts (ATM) and dermis micrografts (DMG) have emerged as promising autologous therapies in regenerative wound care, leveraging mechanically disaggregated cell–matrix constructs to modulate the wound microenvironment and promote tissue repair. This scoping review systematically analyzed clinical studies investigating ATMs and DMGs in acute and chronic wounds. Eight studies, comprising randomized controlled trials, observational studies, and case series, were identified, involving diverse wound types such as burns, ulcers, surgical dehiscence, and posttraumatic defects. All interventions utilized mechanical disaggregation (Rigenera^®^ system) to produce micrografts, which were applied via perilesional injection, scaffold-assisted delivery, or topical administration. Outcomes consistently demonstrated accelerated re-epithelialization, enhanced angiogenesis, improved scar remodeling, and low complication rates. In select studies, micrografts were combined with platelet-rich fibrin or stromal vascular fraction, suggesting potential synergistic effects. While one randomized trial showed superior healing outcomes with DMGs over collagen scaffolds, others yielded mixed results, likely reflecting heterogeneity in methodology and outcome measures. Overall, the available clinical evidence supports the safety, feasibility, and biological activity of micrograft-based therapies. However, larger, standardized, and mechanistically driven studies are required to validate their efficacy and define optimal protocols across wound etiologies.

## 1. Introduction

Wound healing is a dynamic and tightly regulated biological process involving a sequence of overlapping phases—namely inflammation, proliferation, and remodeling—each governed by specific cellular and molecular responses [1]. While most acute wounds progress through this sequence efficiently, chronic wounds, burns, and complex surgical wounds present significant clinical challenges, often resulting in delayed healing, infections, or poor aesthetic and functional outcomes [2]. Chronic and non-healing wounds represent a substantial burden on healthcare systems worldwide, affecting millions of patients and leading to decreased quality of life, prolonged hospitalizations, increased morbidity and significant clinical and financial implications [3]. In Europe, the prevalence and economic impact of chronic wounds—including venous leg ulcers, pressure injuries, and diabetic foot ulcers—are rising, placing sustained demands on healthcare resources [4]. International consensus statements further emphasize the need for early, evidence-based interventions to prevent complications and reduce recurrence [5]. These data underscore the urgency of developing effective, accessible, and biologically driven wound-healing strategies such as micrograft-based therapies. Traditional wound care approaches—such as dressings, debridement, and antibiotic therapy—often fail to address the underlying pathophysiological barriers to healing. As such, there is an urgent clinical need for therapies that not only close wounds but also restore tissue architecture and function [6].

In recent years, regenerative medicine has emerged as a promising field offering innovative solutions to enhance tissue repair and regeneration [7]. Among these, the use of micrografts—particularly those derived from adipose tissue (ATM) and dermal tissue—has gained increasing interest due to their rich cellular and extracellular matrix content, ease of harvest, and potential to modulate the wound microenvironment [8]. Adipose tissue, in particular, contains stromal vascular fractions (SVF), which include mesenchymal stem cells, endothelial progenitor cells, and immune-modulatory factors that contribute to tissue regeneration [9]. Similarly, dermis-derived micrografts (DMG) offer structural proteins, growth factors, and cellular elements that support re-epithelialization and matrix remodeling [10]. These therapies, with their ability to deliver regenerative cell populations and bioactive cues directly to the wound bed, offer a biologically targeted approach that aligns with the principles of personalized and precision medicine. Their application could potentially reduce the need for more invasive procedures, decrease healthcare costs, and improve long-term outcomes for patients with complex wounds [11].

**Figure 1:**
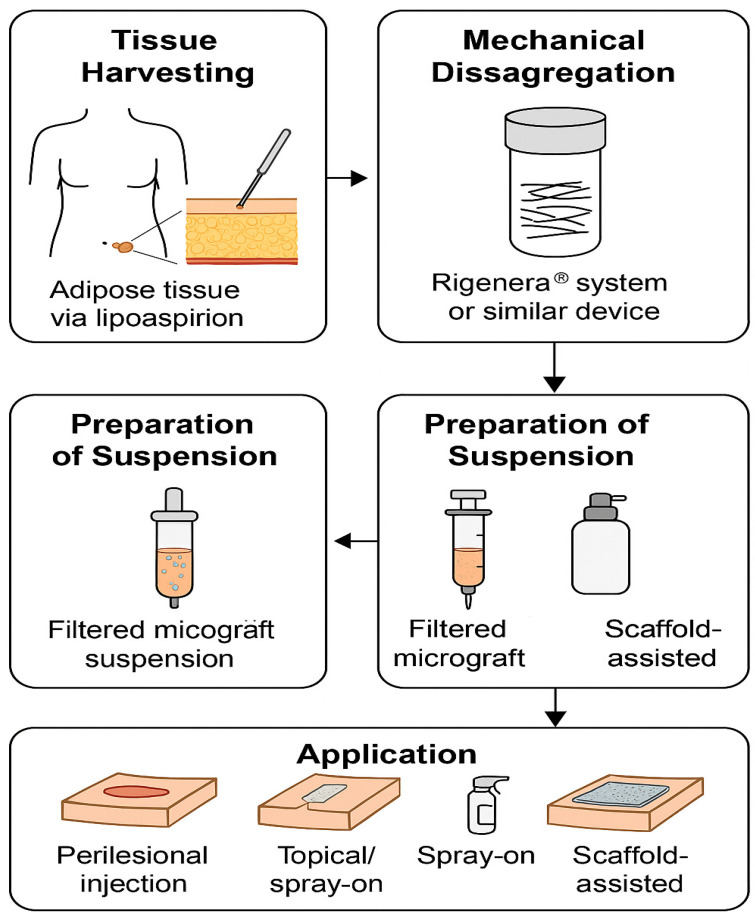
Schematic overview of adipose tissue micrograft (ATM) and dermis micrograft (DMG) preparation. The workflow includes: (1) tissue harvesting—adipose tissue via lipoaspiration and dermal tissue via punch biopsy; (2) mechanical disaggregation using Rigenera^®^ (HBW s.r.l., Candiolo, Italy) and Rigeneracons (HBW s.r.l., Candiolo, Italy) system to produce micrografts while preserving viable cells and extracellular matrix; (3) preparation of the micrograft suspension, including filtration, volume adjustment, and optional combination with adjuncts such as platelet-rich fibrin (PRF) or scaffolds; and (4) clinical application by perilesional injection, topical placement, or scaffold-assisted delivery.

The method of preparation for ATMs and DMGs appears to be a critical determinant of their biological efficacy. Mechanical disaggregation, as performed with systems such as Rigenera^®^ or Rigeneracons, yields micro-fragments of native tissue containing viable cells embedded in their extracellular matrix, thereby preserving cell–cell and cell–matrix interactions essential for regenerative signaling [12,13]. In ATMs, mini-lipoaspiration followed by rapid mechanical processing results in a heterogeneous cell population enriched in mesenchymal stromal cells, pericytes, endothelial progenitors, and adipocytes, along with angiogenic and immunomodulatory cytokines [8,14]. For DMGs, dermal punch biopsies processed in a similar manner produce suspensions containing fibroblasts, keratinocytes, and dermal extracellular proteins such as collagen type I and elastin, which are crucial for re-epithelialization and scar remodeling [15,16]. The preservation of these structural and biochemical components, coupled with the avoidance of enzymatic digestion, is thought to enhance cell viability and reduce processing time compared to enzymatic stromal vascular fraction isolation [17]. Furthermore, the short preparation time (often less than 5 min) and minimal donor-site morbidity facilitate point-of-care application, making these techniques highly adaptable to acute surgical settings and chronic wound management. Future research should aim to standardize preparation parameters—such as tissue volume, disaggregation time, and filtration pore size—to optimize cell yield and functional outcomes. The sequential steps for harvesting, processing, and applying ATMs and DMGs are described in detail (Figure 1).

Several clinical studies have explored the efficacy and safety of these micrografts in promoting wound healing, yet the data remain scattered and heterogeneous in terms of methodology, patient population, wound types, and outcome measures [13,18]. There is a need to systematically consolidate and evaluate the available experimental and clinical evidence to better understand the role, mechanisms, and translational potential of ATMs and dermis micrografts in wound healing. Moreover, preclinical studies in cell cultures and animal models have demonstrated the safety and regenerative potential of mechanically processed adipose and dermal micrografts. However, this review focuses exclusively on human clinical trials, in which dosing, application techniques, and healing outcomes are directly translatable to patient care.

Accordingly, we conducted a scoping review of all published human clinical trials from January 2000 through May 2025 that utilized mechanically processed dermal or adipose micrografts for wound healing.

The aim of this scoping review is to critically assess the current body of literature on the use of adipose tissue and dermis micrografts in wound healing, focusing on clinical outcomes. This review seeks to identify common trends, evaluate the quality of evidence, and highlight areas for future research in regenerative wound care.

## 2. Materials and Methods

### 2.1. Study Protocol

This scoping review was conducted in accordance with the PRISMA Extension for Scoping Reviews (PRISMA-ScR), as developed by Tricco et al. [19]. Prior to initiation, a detailed protocol was drafted to specify objectives, eligibility criteria, search strategy, study selection, data extraction, and synthesis methods.

### 2.2. Information Sources, Literature Search, and Eligibility Criteria

A comprehensive literature search was conducted to identify relevant clinical studies exploring the applications of adipose tissue micrografts (ATM) and dermis micrografts in wound healing. The following electronic databases were systematically searched: PubMed/MEDLINE, Scopus, and Web of Science. The search included studies published from January 2000 to June 2025, and no language restrictions were initially applied. Additional sources were identified by screening the reference lists of included articles and relevant reviews. The search strategy incorporated combinations of Medical Subject Headings (MeSH) and free-text terms such as:

“adipose tissue micrografts”, “dermal micrografts”, “tissue regeneration”, “skin wound healing”, “autologous micrografts”, and “clinical wound repair”. Boolean operators (AND, OR) were used to refine the search.

#### 2.2.1. Inclusion Criteria

This review included clinical studies that investigated the application of adipose tissue micrografts (ATM) or dermis-derived micrografts in the treatment of acute or chronic wounds. Eligible studies encompassed human subjects with wound types such as diabetic ulcers, pressure sores, burns, surgical wounds, and traumatic injuries. Interventions had to involve autologous, mechanically processed, and minimally manipulated tissue preparations, such as micro-fragmented adipose tissue or mechanically disaggregated dermal grafts. Studies were required to report outcomes related to wound healing or tissue regeneration, including but not limited to re-epithelialization, closure rate, angiogenesis, extracellular matrix remodeling, histological regeneration, scar quality, or safety. Only full-text, peer-reviewed articles published in English were considered.

#### 2.2.2. Exclusion Criteria

Studies were excluded if they used enzymatically digested adipose tissue alone (e.g., stromal vascular fraction), isolated stem cells alone, platelet-rich plasma (PRP) alone, or synthetic wound treatments without combination with mechanically prepared adipose micrografts. Combined protocols involving ADSVF and autologous micrografts were considered eligible. Review articles, meta-analyses, editorials, conference abstracts lacking full data, and single case reports were excluded. In addition, studies that did not report any relevant healing or regenerative outcomes, or those that focused solely on molecular mechanisms without functional or morphological endpoints, were not included. Research utilizing non-autologous grafts (e.g., xenogeneic or allogeneic tissue) without a clear emphasis on autologous micrograft-based techniques was also excluded from this review.

### 2.3. Literature Search

The research was conducted by two independent reviewers, who initially examined existing systematic reviews relevant to the topic. However, upon identifying inconsistencies—specifically, a lack of alignment between the stated primary objectives and the studies included in those reviews—the reviewers decided to proceed with a new investigation following the PRISMA-ScR guidelines. The study selection and data collection processes were carried out collaboratively by the two reviewers

### 2.4. Data Collection and Extraction

Data extraction was performed independently by two reviewers, who gathered the following information from each included study: (a) name of the first author, (b) publication year, (c) study design, (d) type of treatment administered, (e) anatomical treatment site, (f) treatment groups, (g) duration of follow-up, and (h) reported outcome measures. Furthermore, comprehensive details were documented, including (a) sample size, (b) surgical technique use and (c) size of the defect. In instances where discrepancies arose between the two primary reviewers, a third reviewer was consulted to resolve disagreements and reach a consensus on data extraction and study eligibility.

## 3. Results

### 3.1. Study Selection Process

A comprehensive literature search was conducted across three electronic databases—Medline (PubMed), Web of Science, and Scopus—resulting in the identification of 102 records. Following the removal of 34 duplicate entries and an additional 6 records excluded for other reasons, 62 studies proceeded to the screening phase. At this stage, 48 studies were excluded due to the absence of a technique description (*n* = 18), in vitro design (*n* = 3), or other unspecified reasons (*n* = 27). The remaining 14 full-text articles were assessed for eligibility, of which 6 were excluded due to inadequate description of outcomes. Consequently, 8 studies fulfilled all inclusion criteria and were incorporated into the final qualitative synthesis.

**Figure 2:**
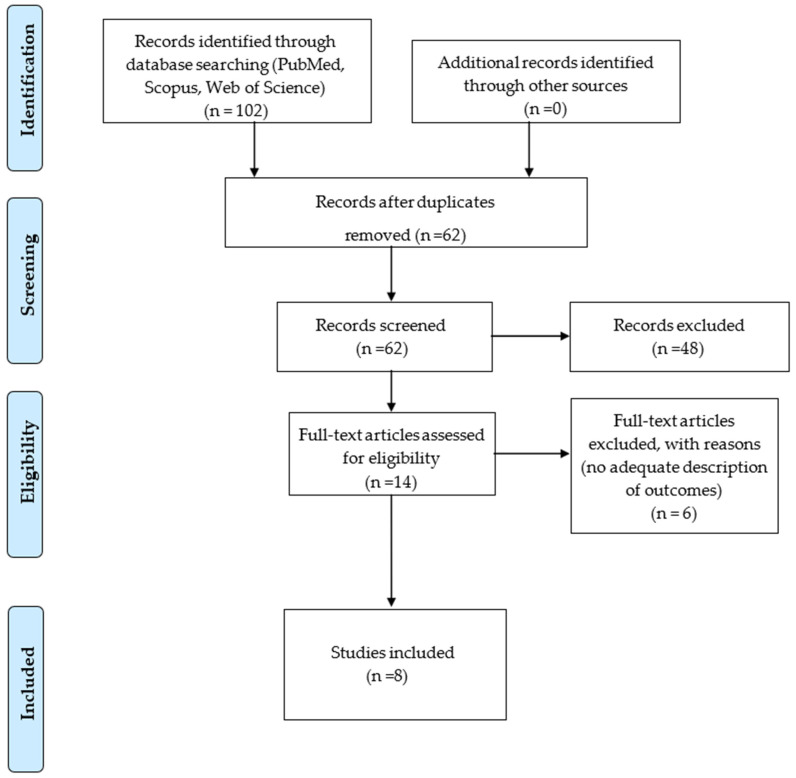
PRISMA 2020 flow diagram for the scoping review process. From: Moher D, Liberati A, Tetzlaff J, Altman DG, The PRISMA Group (2009). Preferred Reporting Items for Systematic Reviews and Meta-Analyses: The PRISMA Statement [20], Modified: The Joanna Briggs Institute Reviewers’ Manual 2015. Methodology for JBI Scoping Reviews. Published by the Joanna Briggs Institute, 2015 [21].

The study selection process is summarized in the PRISMA 2020 flow diagram (Figure 2), adapted from Moher et al. [20] and the Joanna Briggs Institute methodology [21].

### 3.2. Overview of Included Studies and Study Characteristics

Eight clinical studies were identified assessing either adipose-derived micrografts (ATM; *n* = 4) or dermis micrografts (DMG; *n* = 4) in wound healing. Publication years ranged from 2016 to 2024, and sample sizes varied from 3 to 20 patients per study. Designs included case series (*n* = 5), one randomized controlled trial (Tresoldi et al. [22]), one open-label RCT (Iglesias et al. [23]), and one multicenter observational study (Riccio et al. [24]).

### 3.3. Patient Demographics and Wound Types

Patient ages spanned 18–78 years, with a mix of chronic (pressure, diabetic, venous) and acute (surgical dehiscence, burns, post-traumatic) wounds. Among the ATM studies (Table 1 (a–b)), four cohorts totaled approximately 40 patients: small case series in elderly patients with surgical dehiscence (Marcarelli et al. [25]), systemic sclerosis–associated digital ulcers (Iglesias et al. [23]), burn wounds (Andreone et al. [26]), and mixed postsurgical dehiscence (Baglioni et al. [10]). In the DMG group (Table 2 (a–b)), a total of 20 patients were enrolled in the RCT by Tresoldi et al. [22]; the remaining studies did not report precise sample sizes, but addressed hypertrophic/keloid scars (Svolacchia et al. [27]), limb skin defects (Riccio et al. [24]), and mixed chronic ulcers (Miranda et al. [28]). A detailed summary of each trial’s sample size, patient age, micrograft formulation, number of applications and adjunct treatments is provided in Table 3.

Across the six dermal-micrograft (DMG) studies (Riccio et al. [24], Marcarelli et al. [25], Andreone et al. [26], Tresoldi et al. [22], Svolacchia et al. [27], Iglesias et al. [23]) a total of 126 patients (178 wounds) were treated. Patient ages spanned 34–93 years (median ~78 years), and dermal biopsies (0.2–0.5 cm^2^) yielded suspensions of 2.5–5 mL, all delivered in a single session. Adjuncts included collagen sponges (Equine, Integra^®^), PRF, antimicrobial dressings (Acticoat^®^), and NPWT in select cases. Across these DMG trials, reported time to complete epithelial closure ranged from 4 to 12 weeks (median ≈ 8 weeks) with follow-ups of 12–24 weeks (Table 1).

In the three adipose-tissue micrograft (ATM) studies (Baglioni et al. [10], Svolacchia et al. [27], Iglesias et al. [23]), 54 patients (60 digits/wounds) aged 24–80 years (mean ~55 years) received 1.2–10 mL of filtered micrograft or ADSVF preparations, again as a single application alongside standard systemic therapy or HA scaffolds. Median closure occurred at 9 weeks (range 6–14 weeks). Because each study employed different patient populations, micrograft formulations, dosing regimens, outcome definitions, and follow-up schedules, a quantitative meta-analysis was not feasible. Hence, we provide only these descriptive summaries of study characteristics and healing times (Table 2).

### 3.4. Preparation and Application

All studies used mechanical disaggregation (Rigenera^®^/Rigeneracons) to generate micrografts, with two ATM studies additionally combining platelet-rich fibrin (Andreone et al. [26]) or adipose-derived stromal vascular fraction (Iglesias et al. [23]). ATM preparations originated from either subcutaneous fat alone or mixed adipose/dermis. DMG preparations derived exclusively from dermal punch biopsies. Application techniques included:

- Injection: perilesional and intralesional injections (Marcarelli et al. [25], Iglesias et al. [23], Svolacchia et al. [27]).
- Topical/spray-on: onto collagen or synthetic scaffolds (Andreone et al. [26], Riccio et al. [24], Miranda et al. [28]).
- Composite approach: imbibition into dermal substitute plus infiltration (Tresoldi et al. [22]).

### 3.5. Outcomes Measured

Primary endpoints focused on wound closure kinetics and re-epithelialization:

- Time to complete closure ranged from 7–10 days in full-thickness burns (Andreone et al. [26]) up to 90 days for chronic dehiscence (Baglioni et al. [10]).
- Percentage area reduction and closure rates at fixed timepoints (4 weeks in Tresoldi et al. [22]; up to 168 days in Iglesias et al. [23]).

Secondary measures included pain scores (VAS), scar quality (Vancouver scale), neo-angiogenesis, capillary density, and patient-reported hand function or quality-of-life metrics.

In light of the small number of studies and their clinical and methodological heterogeneity, no formal inferential statistical analyses (e.g., *p*-values or confidence intervals) were conducted in this review.

### 3.6. Follow-Up and Safety

Follow-up durations spanned from 4 weeks (Svolacchia et al. [27], Tresoldi et al. [22]) to 1 year (Marcarelli et al. [25]). Adverse events were infrequent and mild: transient donor-site pain or ecchymosis (Iglesias et al. [23]), and one infection leading to dropout (Tresoldi et al. [22]). No serious graft-related complications were reported across the eight studies.

Table 1 presents a detailed overview of clinical studies employing adipose-derived micrografts, whereas Table 2 summarizes investigations involving dermis-derived micrografts, including study methodology, graft application techniques, clinical outcomes, and safety data.

## 4. Discussion

Chronic wounds impose a substantial global burden, affecting an estimated 1–2% of the population in developed countries and contributing significantly to morbidity and healthcare costs [29,30]. The complexity of these wounds often stems from impaired angiogenesis, persistent inflammation, and compromised extracellular matrix remodeling [31]. In this context, regenerative approaches delivering viable cells and matrix components—such as micrograft preparations—are gaining prominence. The mechanotransductive cues preserved in mechanically disaggregated tissue have been shown to modulate cell fate and enhance regenerative capacity [32,33]. Such strategies offer the potential to bridge the gap between conventional wound care and advanced tissue-engineering techniques, warranting further translational investigation.

This scoping review evaluated eight experimental and clinical studies exploring the use of adipose-derived micrografts (ATM) and dermis micrografts (DMG) in wound healing. Together, these investigations encompassed 150 patients and spanned a diverse array of wound types, anatomical regions, and application protocols. ATM approaches—whether paired with platelet-rich fibrin (Andreone et al. [26]) or stromal-vascular fraction (Iglesias et al. [23])—demonstrated rapid healing in burns, surgical dehiscence, and systemic sclerosis–related digital ulcers (closure times as short as 7–10 days, and significant area reductions by 168 days). DMG interventions likewise yielded statistically similar closure rates to controls in post-surgical soft-tissue defects (Tresoldi et al. [22]) and qualitative improvements in scar quality and angiogenesis (Svolacchia et al. [27], Riccio et al. [24]).

The five non-randomized investigations all demonstrated consistently favorable outcomes, however the RCTs provided mixed results. Marcarelli et al. [25] reported significant wound re-epithelialization in surgical wounds treated with micrografts compared to traditional collagen sponges, demonstrating a clear advantage in terms of healing speed and quality. Conversely, the study of Iglesias et al. [23], involving Integra^®^ (Integra LifeSciences, Princeton, NJ, USA) enriched with dermal micrografts, showed limited re-epithelialization, with statistical analysis unable to confirm significant efficacy when compared to controls. Similarly, Andreone et al. [26] demonstrated an enhanced healing process and better scar outcomes when ADSVF combined with adipose micrografts was used alongside conventional medical therapies, suggesting potential synergistic effects.

Although the international literature is now well established in demonstrating the efficacy of adipose-derived micrografts (ATM) and their clear healing effects in preclinical and experimental models, the body of evidence in human studies remains relatively limited [34,35]. For this reason, it became essential to undertake the present review—supplemented by the most recent human studies—to accurately capture and synthesize the clinical data, thereby filling the gap and guiding future research and practice.

Taken together, these studies suggest that both ATMs and dermal micrografts can accelerate wound healing and improve tissue quality across a spectrum of clinical scenarios—from surgical dehiscence and chronic ulcers to burn wounds and systemic-sclerosis–related hand involvement. Notably, one RCT demonstrated clear superiority of Rigenera^®^ micrografts over collagen sponges, while another found no difference, indicating that factors such as graft preparation, dosing, and application technique are likely critical determinants of success. The pain-relieving benefit observed in systemic sclerosis further expands the therapeutic promise of micrografts beyond structural repair.

All of the included trials employed mechanical disaggregation via the Rigenera^®^/Rigeneracons system to generate micrografts—tiny aggregates of autologous cells encased within fragments of their native extracellular matrix [36]. By avoiding harsh enzymatic digestion, this approach preserves critical cell–matrix interactions and enhances cell viability [37]. The process also concentrates progenitor populations (including mesenchymal and endothelial cells) and liberates key growth factors such as VEGF and TGF-β, which drive angiogenesis and granulation tissue formation [10]. Finally, the resulting homogeneous suspension can be delivered uniformly—either by perilesional injection or as a topical spray—ensuring consistent distribution across the wound bed.

Originally developed in the early 2010s for cosmetic and oral-maxillofacial applications (e.g., facial skin rejuvenation, gingival regeneration), the Rigenera^®^ technology has since been adapted for soft-tissue repair. Its key historical advantages over traditional split-thickness grafts are minimal donor-site morbidity, rapid preparation (≤2 min), and the ability to re-treat the same patient multiple times without exhausting donor tissue [38].

Preclinical evidence supports the therapeutic relevance of minimally manipulated adipose-derived preparations in wound healing. In animal burn wound models, adipose tissue-derived stromal vascular fraction (SVF) significantly accelerated healing and enhanced angiogenesis compared to untreated controls, via robust paracrine mechanisms [39]. In rats with acute wounds, both SVF and isolated mesenchymal stem cells reduced wound size and elevated VEGF expression relative to controls, suggesting functional equivalence with enhanced accessibility for SVF [40]. Mechanistic reviews also highlight how non-expanded SVF preserves essential cell–matrix interactions and enables faster, point-of-care preparation—key advantages over enzymatic ADSC protocols [41]. Finally, studies in diabetic mice demonstrate that adipose-derived Muse cells—notably a stem cell-enriched subset—accelerate diabetic skin ulcer healing more effectively than non-Muse cells, integrating into both epidermis and vasculature [muse cells in diabetic wound model [42]. Together, these data underscore that mechanically processed, matrix-preserving adipose preparations—including SVF and micrograft analogs—enhance vascularization, re-epithelialization. Although mechanistic studies were not the primary focus of the included papers, the observed clinical outcomes likely reflect the rich cellular and extracellular matrix content of micrografts. Adipose-derived grafts are known to contain mesenchymal stem cells, pericytes, and cytokines that modulate angiogenesis and immune response [36]. Mechanically disaggregated dermal grafts similarly retain viable fibroblasts and structural proteins, which may synergize to support tissue regeneration. The preservation of native tissue microarchitecture through minimal manipulation may be critical for maintaining biological activity [37].

Application techniques varied widely across studies, including topical placement, scaffold-assisted delivery, and direct injection. Such heterogeneity complicates comparison but also illustrates the adaptability of micrograft strategies to wound type and location. Standardized dosing was largely absent; however, protocols generally adhered to the principle of minimal manipulation to comply with regulatory guidelines and preserve cellular viability. The delivery of micrografts in conjunction with platelet-rich fibrin or collagen scaffolds appears to enhance outcomes, although the contribution of these adjuncts is difficult to isolate.

In direct comparison, adipose-derived micrograft (ATM) studies generally demonstrated more rapid wound closure—particularly in highly vascularized contexts such as burn injuries and dehisced surgical wounds—likely reflecting the potent angiogenic activity of adipose-derived stromal cells. Dermis micrografts (DMG), on the other hand, were especially effective at remodeling scar tissue, yielding notable improvements in collagen organization and scar pliability (as shown by Svolacchia et al. [27]), whereas ATM investigations more often emphasized quantitative wound-size reductions and patient-reported outcomes like pain relief and functional gains. Additionally, the study combining ATMs with platelet-rich fibrin (Andreone et al. [26]) suggested synergistic effects, enhancing both hemostasis and regenerative signaling—an approach that deserves further exploration in future trials.

The addition of adipose-derived stromal vascular fraction (ADSVF) to fat micrografts in systemic sclerosis yielded pain relief and functional improvement, suggesting that micrografts may exert not only regenerative but also anti-inflammatory and neurotrophic effects [23]. Scar remodeling studies indicated long-term aesthetic and functional benefits, including enhanced vascularity and pliability, supporting the concept that micrografts influence wound healing beyond epithelial closure. [13] Additionally, combining micrografts with bioengineered scaffolds, autologous blood products, or advanced delivery systems (e.g., hydrogels or sprays) warrants systematic investigation to maximize therapeutic synergy.

Across all eight studies, complications were rare and mild. In one RCT [23], patients receiving ADSVF-enriched fat micrografts for systemic sclerosis-related hand ulcers experienced only mild donor-site pain and ecchymoses, which resolved within five days9. This adverse effect was also observed in an early phase I trial of stromal Vascular Fraction injection into the fingers of systemic sclerosis patients, where minor bruising and transient finger discomfort were reported [43]. Those findings are consistent with the expected sequelae of lipoaspiration—with no serious safety concerns. In another RCT of dermal micrograft, a single wound infection led to one patient’s dropout; otherwise, no graft-related complications were observed [22]. Girão et al. [44], in a study of striae distensae treated with microneedling plus AMT^®^, advised patients to expect localized redness, swelling, and bruising lasting 2–3 days post-procedure, with no longer-term sequelae reported. No serious adverse events (e.g., graft rejection, systemic reactions) were otherwise reported, supporting the safety of autologous micrografts even in immunocompromised or comorbid populations.

Our findings are consistent with broader evidence synthesized in recent systematic reviews and meta-analyses. In 2020, Gentile and Garcovich [45] reviewed the clinical applications of adipose-derived stem cells in regenerative plastic surgery, reporting enhanced angiogenesis, extracellular matrix remodeling, and anti-inflammatory effects across multiple wound types. Bora and Majumdar [46] summarized stromal vascular fraction biology and translational studies, underscoring the importance of mechanical processing techniques in preserving cell–matrix interactions. Together, these high-level reviews reinforce the translational relevance of mechanically prepared micrografts and support their integration into advanced wound-healing strategies.

Evidence from other surgical specialties further supports the regenerative potential observed in our wound-healing findings. In oral and maxillofacial surgery, mechanically prepared periosteum- or dental pulp–derived micrografts combined with scaffold materials such as collagen or PLGA/HA have demonstrated improved clinical and histological outcomes in bone regeneration. Randomized controlled trials have reported significant gains in clinical attachment level and radiographic bone fill in periodontal intrabony defects treated with dental pulp-derived micrografts compared with conventional therapy [47]. Similarly, in sinus lift augmentation, periosteum-derived micrografts incorporated into collagen membranes enhanced new bone formation and integration compared with scaffold alone [48]. Narrative reviews have emphasized that the mechanical disaggregation process preserves native extracellular matrix components and cellular niches, potentially explaining the observed regenerative benefits [38]. In orthopedics, microfragmented adipose tissue (MFAT) obtained via minimal mechanical processing has been investigated for intra-articular application in knee osteoarthritis. Randomized trials comparing MFAT to platelet-rich plasma (PRP) have shown comparable clinical improvements at one year [49], while more recent data confirm similar efficacy profiles over mid-term follow-up [50]. Collectively, these cross-disciplinary findings align with our clinical results, reinforcing the concept that micrografts retaining structural and cellular integrity can promote tissue repair, with outcomes influenced by tissue target, delivery method, and supportive scaffold use.

Despite its strengths, this study is subject to several limitations that may affect the interpretation and generalizability of the findings. Although studies using ADSVF alone were excluded, one included tria [23]; combined ADSVF with mechanically processed adipose micrografts, which met our inclusion criteria. The current body of literature on adipose-derived and dermis micrografts in wound healing is characterized by considerable heterogeneity, with study designs spanning small case series, retrospective observational reports, and only a few randomized controlled trials. This variability in methodology, coupled with a wide range of wound types and patient populations, makes direct comparison of outcomes challenging. Furthermore, several studies failed to report key details such as exact sample sizes, the duration of follow-up, or the use of standardized outcome measures, creating gaps in our understanding of treatment efficacy. Most trials monitored patients for six months or less, leaving unanswered questions about the long-term durability of regenerated tissue, including scar remodeling and functional resilience. Finally, the predominance of open-label designs and the limited number of blinded, controlled comparisons raise concerns about potential bias in subjective endpoints such as pain relief and quality-of-life assessments.

All identified studies reported favorable outcomes, raising the possibility of publication bias. The absence of published negative or null results may reflect selective reporting or non-publication of unfavorable findings. Moreover, the lack of registered, prospectively designed trials limits the ability to assess the presence of unpublished data. This underscores the need for trial registration and comprehensive reporting of all outcomes—positive or negative—in future studies to ensure a more balanced evidence base.

To strengthen the evidence base for micrograft therapies, future research should prioritize the adoption of core outcome sets—such as those recommended by the TIME-Wound Healing Council—to ensure consistency and comparability across studies. Mechanistic and dose–response trials that systematically vary micrograft concentration, volume, and the inclusion of co-factors like platelet-rich fibrin or scaffold materials will be essential for defining optimal treatment protocols. Exploring combination strategies that integrate micrografts with emerging biomaterials (for example, injectable hydrogels or nanofiber meshes) and adjunctive modalities (such as low-level laser therapy or tailored growth-factor cocktails) may further enhance regenerative outcomes. Finally, large-scale, multicenter randomized controlled trials with adequate blinding and statistical power are needed to confirm both the clinical efficacy and cost-effectiveness of micrograft approaches, particularly for chronic, non-healing ulcers that pose the greatest therapeutic challenge.

## 5. Conclusions

In summary, the body of preclinical and early clinical evidence strongly supports the safety and regenerative capacity of adipose-derived and dermis micrografts prepared via mechanical disaggregation (Rigenera^®^/Rigeneracons). Across studies that were selected, these micrografts have consistently accelerated re-epithelialization, enhanced angiogenesis, and improved scar quality with minimal donor-site morbidity or serious adverse events. Their ability to preserve native extracellular matrix, concentrate progenitor cells, and deliver a rich paracrine milieu distinguishes this point-of-care technology from enzymatic or cell-expansion approaches, offering a rapid, cost-effective adjunct to standard wound care.

Despite these promising results, the clinical literature remains limited by small sample sizes, heterogeneous study designs, and relatively short follow-up. Larger, multicenter randomized trials—ideally incorporating standardized outcome measures and blinded assessments—are needed to confirm efficacy across diverse wound types and patient populations. Future research should also explore optimized dosing, combination with bioengineered scaffolds or growth-factor adjuvants, and long-term functional outcomes to fully define the place of micrografting within the regenerative medicine armamentarium. As the evidence base matures, adipose and dermis micrografts have the potential to shift from an experimental niche to a mainstream therapeutic option for both acute and chronic wound healing.

## Figures and Tables

**Table 1:** Clinical studies on adipose-derived micrografts (ATM) in wound healing: Study characteristics, patient demographics, wound types, preparation method, application technique, outcomes, and safety.

Author, Year	Study Design	Patient Demographics	Wound Types	Graft Preparation Method	Application Technique	Outcomes Measured	Follow-Up Duration	Adverse Events
Marcarelli et al. [25] (2017)	Case series (3 patients)	3 elderly (61–78 years old); HTN, DM, MS	Surgical wound dehiscence	Rigeneracons mechanical disaggregation of 1 cm^2^ skin in 1 mL saline over 90–120 s	Perilesional injection & application onto equine collagen sponge scaffold	Time to complete closure (mean 30 days); wound area reduction; re-epithelialization	Weekly up to 1 year	None
Andreone et al. [26] (2019)	Retrospective case series (5 patients)	2 massive burns; 3 chronic burn wounds	Full-thickness burns	Rigeneracons micrografts + autologous PRF	Spray-on via Vivostat^®^ Spraypen (Vivostat A/S, Alleroed, Denmark)onto Integra^®^ dermal template; antimicrobial dressing	Time to re-epithelialization (7–10 days); rate of complete graft incorporation	Mean 7–10 days	None reported
Iglesias et al. [23] (2023)	Open-label RCT (20 SSc patients)	>18 y, BMI > 18 kg/m^2^	Digital ulcers, Raynaud’s	ADSVF via collagenase digestion + fat micrograft; 60 mL lipoaspirate → 3 mL ADSVF → mixed with 40 mL fat	Injection along radial/ulnar digital pedicles and subcutaneously into palm and dorsum	Time to complete closure (all wounds were closed by week 9), Pain VAS; ulcer count; Raynaud’s frequency/intensity; mobility; thumb opposition; capillary density; hand-function; QoL scores	0 & 168 days	Donor site pain; ecchymosis (resolved day 5)
Baglioni et al. [10] (2024)	Uncontrolled pilot (12 patients)	Patients with postsurgical dehiscence	Chronic wound dehiscence	Rigenera^®^ mechanical micrografts from adipose ± dermis (fluid suspension)	Injection into wound edges and floor under local/regional block	% lesion size reduction; % complete healing (75% of wounds achieved full closure by day 90 (and 91.6% had ≥ 50% reduction by day 90) cellular antioxidant activity; exosome profiling	90 days	Not reported

ATM: adipose tissue micrograft; PRF: platelet-rich fibrin; ADSVF: adipose-derived stromal vascular fraction; QoL: quality of life; VAS: visual analogue scale; HTN: hypertension; DM: diabetes mellitus; MS: multiple sclerosis. All micrografts were prepared using mechanical disaggregation systems (Rigenera^®^ or Rigeneracons). Sample sizes reflect available data from each study; some were approximated where exact numbers were not explicitly stated.

**Table 2:** Clinical studies on dermis-derived micrografts (DMG) in wound healing: Study characteristics, patient demographics, wound types, preparation method, application technique, outcomes, and safety.

Author, Year	Study Design	Patient Demographics	Wound Types	Graft Preparation Method	Application Technique	Outcomes Measured	Follow-Up Duration	Adverse Events
Svolacchia et al. [27] (2016)	Case series (n not stated)	Not reported	Hypertrophic & keloid scars	Rigenera™ mechanical disaggregation of dermal punch biopsies (3 mm) into saline suspension	Intralesional injection	Scar appearance & texture (Vancouver scale); histology (papillary dermis architecture; collagen realignment)	4 months	None
Miranda et al. [28] (2018)	Case series (*n* = 15 patients)	Age: mean 72.2 ± 8.41 years (range 57–82)	Chronic ulcers (venous, diabetic, pressure, post-traumatic)	Rigeneracons dermal micrografts (collected with a 3 mm diameter punch biopsy)	Topical application on wound bed	Early response, time to complete healing (20–160 days); granulation tissue formation; scar quality neo-angiogenesis	6 months	No complication noticed. One patient died of cardiovascular causes after 16 weeks (unrelated to procedure)
Tresoldi et al. [22] (2019)	RCT (23 units; 20 patients)	median 78 years; 4 F/16 M	Acute postsurgical soft-tissue loss (BCC, SCC, others)	Rigeneracons-derived dermal micrografts in 2.5 mL saline over 90 s	Imbibed into Integra^®^ dermal substitute + perilesional infiltration	Re-epithelialization rate (%) at 4 weeks	4–6 weeks	1 wound infection → dropout
Riccio et al. [24] (2019)	Multicentre observational	Not reported	Full-thickness posttraumatic limb skin defects	Rigeneracons mechanical micrografts	Spray-on micrograft suspension over wound bed	Time to closure (average 48 days, range 35–84); quality of regeneration (clinical assessment)	Not reported	No complication noticed

DMG: dermis-derived micrograft; BCC: basal cell carcinoma; SCC: squamous cell carcinoma; QoL: quality of life; VAS: visual analogue scale. All studies employed mechanical disaggregation of dermal tissue (Rigenera^®^ or Rigeneracons, Rigenera HBW, Candiolo, Italy). “n not reported” indicates studies where sample size or demographic data were not specified. One patient death ([28]. was unrelated to the intervention.

**Table 3:** Summary of each study.

Study (Citation)	Number of Patients (n)	Patient Age (Mean ± SD or Range)	Micrograft Volume/Formulation	Number of Applications	Adjunct Treatments
Iglesias et al. [23] (2023)	20	35–72 yrs; mean ≈ 54 yrs	ADSVF + fat micrografts (volume: 10 mL injected into hand)	Single session	Standard wound dressings
Baglioni et al. [10] (2024)	14	24–80 yrs (mean ~55 yrs; mode 71–80 yrs)	Fat micrografts: 10 mL lipoaspirate processed 3 min @80 rpm; Dermis micrografts: skin fragment (~lesion/20) in 6 mL saline	Single application	Collagen scaffold + hydrofibre/polyurethane foam dressings
Riccio et al. [24] (2019)	70	53 yrs (34–74 yrs)	1 cm^2^ dermal biopsy → mechanical disaggregation in two 3 mL saline aliquots; yield ~5 mL suspension	Single application	Equine collagen sponge “biocomplex,” paraffin gauze dressing
Andreone et al. [26] (2019)	5	22–46 yrs	4 × 2 cm^2^ dermal sample (0.2 mm thick) mechanically disaggregated → 5 mL cell suspension; PRF: 5 mL obtained from 120 mL autologous blood	1–3 injections per patient (case-dependent)	Integra^®^ dermal template; Acticoat^®^ antimicrobial dressing (Smith & Nephew Medical Ltd., Hull, UK); NPWT in one case
Marcarelli et al. [25] (2017)	3	Elderly —not specified	Autologous micro-grafts via Rigeneracons (filtered <80 µm) suspended in saline (volume NR)	1–2 applications per patient	NR
Miranda et al. [28] (2018)	15	(mean ± standard deviation) 72.2 ± 8.41	Specimen collected with a 3 mm diameter biopsy punch and dissociated by the Rigenera System	Simple application	NR
Tresoldi et al. [22] (2019)	20 (24 wounds)	NR	Fat micrografts + adipose-derived SVF injections into fingers (volume NR)	Single application	+standard systemic sclerosis medical therapy
Svolacchia et al. [27] (2016)	14	41–58 yrs	1 mL viable adipose micrografts (50 µm-filtered) emulsified in 1 mL cross-linked hyaluronic acid scaffold; lipoaspirate: 3 mL yield ≈1.2 mL final suspension	Single application	Panthenol “Dermal plus 25 High Performance” (Beauty System Pharma S.r.l., Padova, Italy) cross-linked HA; no other adjuncts

ADSVF = Adipose-Derived Stromal Vascular Fraction; ATM = Adipose Tissue Micrograft; DMG = Dermis Micrograft; PRF = Platelet-Rich Fibrin; HA = Hyaluronic Acid; NPWT = Negative Pressure Wound Therapy; NR = Not Reported. Micrograft volumes and formulations are based on the data explicitly reported in each publication. Where precise quantities were unavailable, approximate volumes or qualitative descriptions were included. All applications were performed using mechanical disaggregation (Rigenera^®^ or Rigeneracons) unless otherwise specified.

## Data Availability

Available upon request on corresponding author.

## Abbreviations

The following abbreviations are used in this manuscript:

ADSVF	Adipose-Derived Stromal Vascular Fraction
ATM	Adipose Tissue Micrograft
BCC	Basal Cell Carcinoma
BMI	Body Mass Index
DM	Diabetes Mellitus
DMG	Dermis Micrograft
F	Female
HTN	Hypertension
Integra^®^	Integra^®^ Dermal Regeneration Template (Integra LifeSciences, Princeton, NJ, USA)
M	Male
MS	Multiple Sclerosi
PRF	Platelet-Rich Fibrin
PRISMA-ScR	Preferred Reporting Items for Systematic Reviews and Meta-Analyses Extension for Scoping Reviews
QoL	Quality of Life
RCT	Randomized Controlled Trial
SCC	Squamous Cell Carcinoma
SSc	Systemic Sclerosis
SVF	Stromal Vascular Fraction
TGF-β	Transforming Growth Factor Beta
VAS	Visual Analogue Scale
VEGF	Vascular Endothelial Growth Factor
